# Cardiomyocyte‐enriched OTUD5 alleviates septic cardiomyopathy by promoting NLRP3 deubiquitination and inhibiting NLRP3 inflammasome activation

**DOI:** 10.1002/ctm2.70743

**Published:** 2026-07-12

**Authors:** Yucheng Jiang, Zhihan Jia, Zhaozheng Zheng, Yingjie Liao, Qingqing Zhao, Wante Lin, Diyun Xu, Naijin Zhang, Guang Liang, Bozhi Ye

**Affiliations:** ^1^ Department of Geriatric Medicine First Affiliated Hospital of Wenzhou Medical University Wenzhou Zhejiang China; ^2^ Chemical Biology Research Center School of Pharmaceutical Sciences Wenzhou Medical University Wenzhou Zhejiang China; ^3^ Department of Cardiology and the Key Laboratory of Cardiovascular Disease of Wenzhou the First Affiliated Hospital, Wenzhou Medical University Wenzhou Zhejiang China; ^4^ Department of Cardiology First Hospital of China Medical University Shenyang City China; ^5^ School of Pharmaceutical Sciences Hangzhou Medical College Hangzhou Zhejiang China

**Keywords:** deubiquitinating enzyme, NLRP3, OTUD5, pyroptosis, septic cardiomyopathy

## Abstract

**Background:**

Septic cardiomyopathy (SCM) is the leading cause of mortality among patients diagnosed with sepsis. Nevertheless, the precise mechanisms underlying its pathogenesis remain poorly understood. Deubiquitinating enzymes (DUBs) play a vital role in various cardiovascular diseases.

**Methods:**

This study investigated deubiquitinating enzymes in septic myocardial injury via public RNA‐Seq. Pyroptosis was modelled in primary neonatal rat cardiomyocytes using lipopolysaccharide (LPS) and nigericin, with levels assessed by IL‑1β ELISA, Western blot, PI staining, CCK‑8 and LDH assays. Potential substrates of OTUD5 were screened by Co‑immunoprecipitation. Cardiomyocyte‑specific OTUD5‑knockout mice generated by CRISPR/Cas9 were subjected to LPS‑ or Caecal ligation and puncture (CLP)‑induced sepsis models for cardiac function and pyroptosis evaluation. AAV9‑mediated cardiomyocyte‑specific OTUD5 overexpression in NLRP3‑knockout mice was used to validate OTUD5‑NLRP3 functional interaction.

**Results:**

This study identifies the deubiquitinating enzyme OTUD5 as being significantly upregulated in myocardial tissue subjected to sepsis induced by LPS and CLP. Cardiomyocyte‐specific knockout of OTUD5 leads to exacerbated septic myocardial injury and pyroptosis. Mechanistically, OTUD5 directly interacted with NLRP3, with its C224 site facilitating deubiquitination to inhibit NLRP3 activation. Notably, the protective effects associated with OTUD5 overexpression were lost in NLRP3 knockout mice, underscoring its dependence on NLRP3 for function.

**Conclusions:**

This study has confirmed that OTUD5 inhibits pyroptosis by suppressing the activity of NLRP3, thereby ameliorating septic cardiomyopathy. OTUD5 is worthy of further exploration as a potential therapeutic target for septic cardiomyopathy.

**Key points:**

- The expression levels of the deubiquitinating enzyme OTUD5 are elevated in tissues affected by sepsis‐induced cardiomyopathy.
- Cardiomyocyte‐specific OTUD5 is a protective factor against myocardial pyroptosis and cardiac dysfunction induced by LPS and CLP.
- OTUD5 interacts with NLRP3, and its C224 site promotes deubiquitination while inhibiting the activation of NLRP3.

## Graphical abstract

A schematic representation of cardiomyocyte‐enriched OTUD5 alleviates septic cardiomyopathy by promoting NLRP3 deubiquitination and inhibiting NLRP3 inflammasome activation.

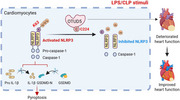

## 1 INTRODUCTION

Sepsis, defined as lethal organ damage caused by the host's inappropriate response to infection, is still the foremost killer of critically ill patients.^1^ Research indicates that approximately 50% of sepsis patients develop cardiomyopathy,^2^ with the mortality rate closely associated with both systolic^3^ and diastolic^4^ dysfunction, highlighting the critical relationship between cardiac function and patient prognosis. Notably, cardiac performance often normalizes within 2 weeks following sepsis, suggesting a primarily functional rather than structural pathological mechanism.^5^ Despite significant advancements in intensive care and anti‐infective therapies, the pathogenesis of septic cardiomyopathy (SCM) is still not fully understood, and there is currently a lack of effective targeted therapy.^6^ Consequently, it is imperative to conduct thorough investigations into the molecular mechanisms underlying septic cardiac dysfunction and explore novel therapeutic targets to enhance patient outcomes and reduce mortality rates.

Pyroptosis is a gasdermin‐dependent, programmed cell death process.^7^ Emerging evidence has implicated it in the pathogenesis of SCM.^8^ The receptor TREM‐1 has been shown to promote septic myocardial pyroptosis and contribute to SCM.^9^ In addition, Cortistatin inhibits cardiomyocyte pyroptosis through the SSTR2‐AMPK‐NLRP3 signalling pathway, providing a protective effect against SCM.^8^ Moreover, m6A modification of SOX18 enhances PTX3 expression and increases cardiomyocyte pyroptosis in sepsis‐induced cardiomyopathy.^10^ These findings reveal pyroptosis as a key driver underlying myocardial injury in SCM. This suggests that pivotal mediators of pyroptosis could serve as promising diagnostic, prognostic and therapeutic targets. While specific agents aimed at these crucial pyroptotic proteins have been reported in literature, none are currently employed clinically for SCM treatment. This highlights an urgent need for developing targeted therapies based on these novel insights.

The role of post‐translational modifications (PTMs), especially deubiquitination, in cardiovascular diseases is increasingly acknowledged.^11^, ^12^ OTUD5, a member of the OTU deubiquitinase family, modulates various cellular processes through the deubiquitination of key proteins and is associated with immunity,^13^ DNA repair, cancer,^14^ inflammation, genetic disorders^15^ and cardiomyopathy.^16^ OTUD5 has been demonstrated to facilitate endothelial ferroptosis during the progression of septic cardiomyopathy.^17^ Mutations in human OTUD5 have been linked to developmental defects and infant mortality resulting from sepsis.^18^ However, its function in SCM and potential association with pyroptosis remains unexplored.

The present study seeks to illuminate the protective mechanisms of the deubiquitinating enzyme OTUD5 in SCM, thereby offering new theoretical insights and potential therapeutic targets.

## 2 MATERIALS AND METHODS

### 2.1 Animal experiment

Myocardium‐specific OTUD5 gene knockout (OTUD5^fl/y^Myh6‐Cre, OTUD5CKO) mice and global NLRP3 gene knockout (NLRP3^−/−^) mice (strain number: T010873) were generated on a C57BL/6J background by Jicui Company. Wild‐type C57BL/6J mice were purchased from Wenzhou Huihong Medical Devices Co., Ltd. All experimental animals were male, specific pathogen‐free‐grade, 8 weeks of age, averaging 21 ± 1 g in body weight. Mice were housed in a controlled SPF facility under constant ambient temperature, with relative humidity maintained at 50% year‐round and a 12‐h lighting cycle. And meloxicam (single dose 5 mg·kg^−^^1^ subcutaneous injection) was given after the operation. Euthanasia was carried out in accordance with the AVMA 2020 guidelines, first inducing deep anaesthesia with 5% isoflurane and then performing cervical dislocation. The Animal Experiment Ethics Committee of Wenzhou Medical University approved all animal procedures (Ethical Approval No.: wydw2024‐0164).

(1) Establishment of the LPS‐induced murine model of septic cardiomyopathy: On the day prior to modelling, mice were fasted and deprived of water for 6 h before being weighed. Lipopolysaccharide (LPS) solution was freshly prepared and administered intraperitoneally at a dose of 10 mg·kg^−^^1^ body weight; control animals received an equivalent volume of 0.9% normal saline. At 12–24 hours post‐LPS injection, mice exhibited characteristic signs of systemic illness, including limb weakness, lethargy, hunched posture and tremors. Cardiac function was subsequently assessed via transthoracic echocardiography, followed by euthanasia and collection of cardiac tissue and serum samples. Experimental groups: OTUD5^fl/y^ + saline group, OTUD5CKO + saline group, OTUD5^fl/y^ + LPS group, OTUD5CKO + LPS group (*n* = 6).

(2) Caecal ligation and puncture (CLP)‐induced murine model of septic cardiomyopathy: After 1 week of acclimatization, mice fasted 12 h before surgery. Using isoflurane inhalation anaesthesia, with an induction concentration of 3% and a maintenance concentration of 2%, and continuous administration through a small animal anaesthesia machine. The abdominal region was disinfected with 70% ethanol, and aseptic surgical techniques were employed. A midline incision of approximately 1 cm was made in the abdominal wall. The caecum was exteriorized using forceps, and one‐third of its distal portion was ligated with a 3‐0 non‐absorbable suture. A 25‐gauge needle was used to puncture the ligated cecum once, allowing extrusion of a small amount of faecal material, which was then repositioned. The peritoneal wall was closed with interrupted layer sutures, followed by immediate subcutaneous injection of warm saline for fluid therapy. Experimental groups: OTUD5^fl/y^ + sham group, OTUD5CKO + sham group, OTUD5^fl/y^ + CLP group, OTUD5CKO + CLP group (*n* = 6).

(3) Generation of the AAV9‐mediated cardiac gene delivery model: Mice were injected via the tail vein. Prior to injection, the tail was wiped with alcohol to disinfect and dilate the vasculature. The AAV9 viral suspension was diluted in sterile normal saline or PBS to the desired working titre. We loaded 100 µL of virus into a 1‑mL syringe, warmed and straightened the tail, and injected into the distal lateral vein. The viral solution was injected slowly to avoid extravasation. Upon completion, the injection site was compressed with a cotton swab for 1 min to prevent bleeding, after which the animal was returned to its cage. Experimental groups: WT + AAV9‐cTnT‐EV + CLP group, WT + AAV9‐cTnT‐OTUD5^oe^ + CLP group, NLRP3^−/−^ + AAV9‐cTnT‐EV + CLP group, NLRP3^−/−^ + AAV9‐cTnT‐OTUD5^oe^ + CLP group (*n* = 6).

### 2.2 Echocardiography

One day before imaging, mice were depilated on the thoracoabdominal area. Mice were anesthetized with 1%–2% isoflurane (R510‐22‐10, Ruiwode Life Science) and imaged using high‐frequency echocardiography to obtain long and short axis images of the parasternal left ventricle. Echocardiographic parameters, including left ventricular internal diameter (LVIDd/s) and wall thickness (LVAWd/s, LVPWd/s, IVSd/s), were measured and analysed using Vevo LAB software (FUJIFILM Visual Sonics) to evaluate cardiac structure and function.

### 2.3 Serum biochemical analysis and measurement of inflammatory cytokines

Whole blood samples should be left to stand at room temperature for 1 h, and then centrifuged at 1000 × g for 20 min. The supernatant was carefully collected, ensuring no haemolysis or contamination. The lactate dehydrogenase (LDH) content was measured using a commercial LDH assay kit (BC0685, Solarbio). Creatine kinase‐MB (CK‐MB) levels were determined using a CK‐MB assay kit (E006‐1‐1, Nanjing Jiancheng Bioengineering Institute). Cardiac troponin T (cTnT/TNNT2) concentrations were quantified using a mouse cTnT enzyme‐linked immunosorbent assay (ELISA) kit (E‐EL‐M1801c, Wuhan Elabscience Biotechnology Co., Ltd.).

### 2.4 Histopathological evaluation

We stained myocardial paraffin sections with hematoxylin and eosin (H&E) (G1120, Solarbio) following standardized protocols to systematically evaluate myocardial tissue morphology and pathological alterations. In addition, cardiac cryosections were processed for apoptosis TUNEL staining (E‐CK‐A322, Elabscience Biotechnology) according to the accurate protocol, enabling reliable identification and quantitative analysis of apoptotic cells through a standardized experimental procedure.

### 2.5 Immunofluorescence

Frozen 5‑µm heart sections were rinsed with PBS to remove residual OCT (4583, Biosharp). Fully immerse the tissue sections in 4% paraformaldehyde for fixation for 10 min, then wash with PBS. Perform permeabilization using 0.5% Triton X‐100, then wash again with PBS. Block nonspecific binding sites by incubating the sections with 5% bovine serum albumin (BSA, A1933, Merck) at 37°C for half hour, then wash with PBS. Apply the primary antibodies including anti‑Vimentin (mouse monoclonal, Cat# ab8978, 1:200, Abcam), anti‑α‑SMA (mouse monoclonal, Cat# 48938, 1:200, Cell Signaling Technology), anti‑CD68 (mouse monoclonal, Cat# ab201973, 1:150, Abcam) and anti‑OTUD5 (rabbit polyclonal, Cat# 21002‑1‑AP, 1:200, Proteintech) uniformly onto the tissue sections and incubate in a humidified chamber at 4°C. Next, warm the sections at 37°C for 1 hour. Prior to secondary antibody application, remove the primary antibody solution by washing with PBS buffer. Add fluorescently labelled secondary antibodies evenly to the sections, ensuring species‐specific detection (e.g., anti‐mouse and anti‐rabbit secondary antibodies used as appropriate), and incubate at 37°C for 2 h in darkness. Subsequently, apply an appropriate volume of mounting medium containing DAPI (Cat no. 0100–20, SouthernBiotech). Visualize and capture images using confocal laser scanning microscopy.

### 2.6 Cell culture and transfection

NIH/3T3 cells (Cell Bank of the Chinese Academy of Sciences) were cultured in high‐glucose DMEM (Gibco, Germany) containing 10% FBS (R223‐00, Vazyme), within a humidified incubator at 37°C and 5% CO_2_. HL‐1 cells (Cell Bank of the Chinese Academy of Sciences) were cultured in Claycomb media (51800C, Sigma) that needs to be supplemented by norepinephrine (A0937, Sigma), FBS and 4 mM L‐Glutamine (G0200, Solarbio). Neonatal rat primary cardiomyocytes (NRCMs) were isolated from Sprague–Dawley rats within 72 h after birth. Hearts were quickly digested with trypsin (Gibco) through sequential 8‐min cycles at 37°C. Cells were collected by centrifugation, resuspended and purified via differential adhesion. Cardiomyocytes were cultured in high‐glucose DMEM supplemented with 10% fetal bovine serum (FBS, R223‐00, Vazyme), 0.1 mM 5‐bromodeoxyuridine (5‐BrdU, HY‐15910, MedChemExpress), and penicillin/streptomycin. All cell lines and primary cells were maintained at 37°C with 5% CO_2_ in a sterile, humidified chamber. Sterile technique was used throughout in a biosafety cabinet.

Mice were intraperitoneally injected with 6% starch broth one day prior to isolation. At the time of experiment, mice were anaesthetized with isoflurane and sacrificed by cervical dislocation. The peritoneal cavity was lavaged with RPMI 1640 medium (C11875500BT, Gibco), and the lavage fluid was repeatedly collected. The recovered fluid was centrifuged to pellet primary peritoneal macrophages, which were then resuspended and plated for further experiments.

Silencing the OTUD5 genes by small interfering RNA (siRNA; si‐rat‐OTUD5, siG2012030313379004, RIBOBIO). Silencing of these genes were achieved by Lipo2000 (Thermo Fisher Scientific). Expression plasmids Flag‐OTUD5‐WT (Mouse and Rat), Flag‐OTUD5‐C224A (Mouse), HA‐His‐NLRP3‐WT (Mouse), HA‐His‐NLRP3‐PYD (Mouse), HA‐His ‐NLRP3‐LRR (Mouse), HA‐His‐NLRP3‐NACHT (Mouse), HA‐Ub, HA‐K48Ub, and HA‐K63Ub (Mouse) plasmids were obtained from Genechem. Expression plasmid was transfected with Lipo3000 (L3000015, Thermo Fisher Scientific).

### 2.7 Western Blot

Protein samples were loaded and run. Proteins were blotted onto PVDF (IPVH00010, Merck) in ice‑cold transfer buffer (300 mA, 90 min). The membrane was blocked with 5% skim milk (1172GR500, Biofroxx) at room temperature for 1 h, washed with TBST, and then incubated overnight at 4°C with the primary antibody. After washing with TBST, the membrane was incubated with the corresponding secondary antibody at room temperature for 1 h, washed again, and then treated with chemiluminescent reagents (ECL, P10300, NCM) before chemiluminescent detection.

Antibodies against OTUD5 (21002‐1‐AP,1:1500), anti‐HA (M20003S,1:1500), anti‐Flag (20543‐1‐AP,1:1500), anti‐His(66005‐1‐IG,1:1500) and normal rabbit IgG (B900610) were procured from Proteintech. Antibodies against GSDMD (ab215203,1:1000) were procured from Abcam. Antibodies against anti‐Caspase‐1 (sc‐56036,1:200) were procured from Santa Cruz Biotechnology. Antibodies against GAPDH (5174,1:1000) were obtained from CST. Anti‑NLRP3 antibody (AG‐20B‐0014‐C100,1:1000) were procured from Adipogen.

### 2.8 Co‐Immunoprecipitation (Co‐IP)

Protein samples were extracted from cells and tissues, with a portion of the protein supernatant retained as Input. The remaining supernatant was incubated with corresponding primary antibodies at 4°C overnight on a rotator, with negative controls included. Antibody‑incubated samples were mixed with Protein A + G agarose beads (P2012, Beyotime) and rotated at 4°C for 4 h for complex formation. After centrifugation, the magnetic beads were retained and the supernatant discarded. PBS‑washed beads were resuspended in loading buffer and heated for 10 min at 100°C in a metal bath.

### 2.9 Assessment of Interleukin‐1β (IL‐1β), interleukin‐18 (IL‐18), cell viability, Lactate dehydrogenase (LDH) release and apoptosis

Tissue/cell culture IL‑1β (F10770, Shanghai Xitang Biotech) and IL‑18 (E‐EL‐M0730, Elabscience Biotechnology) were quantified via ELISA kits. Apoptosis, proliferation and cytotoxicity were assessed by Hoechst 33342/PI staining (CA1120) and CCK‑8 assay (C0038, Dojindo).

### 2.10 Real‐time quantitative PCR (RT‐qPCR)

Total RNA was isolated with TRIzol (15596018, Thermo Fisher) reagent under low‐temperature conditions to preserve RNA integrity. The isolated high‐quality RNA was subsequently reverse‐transcribed into complementary DNA (cDNA) using the HiScript III All‐in‐One RT SuperMix Perfect for qPCR kit (Cat. No. R333‐01, Vazyme) following the protocol. The resulting cDNA was then precisely quantified and used to prepare the optimized PCR reaction mixture with the SYBR qPCR Master Mix, and RT‐qPCR was performed using a rigorously validated pre‐set thermal cycling program with appropriate controls. Table S1 lists the primer sequences used in this study.

### 2.11 Bioinformatic analysis of public RNA‐seq datasets

Public transcriptomic datasets relevant to septic cardiac injury were downloaded from Gene Expression Omnibus (GEO) via terms ‘LPS’, ‘CLP’, and ‘heart’. Two datasets containing both LPS‑induced (GSE200267) and CLP‑induced (GSE200171) murine myocardial samples alongside sham controls were selected. Normalized gene expression matrices were downloaded for each dataset. The expression values of deubiquitinating enzyme (DUB) genes were extracted, and expression differences between sepsis and controls were analysed, applying criteria of |log_2_ fold change| > .585 and adjusted p‑value < .05. The analysis specifically focused on the OTU family of deubiquitinases.

### 2.12 Identification of OTUD5‐interacting proteins by LC–MS/MS

To screen for OTUD5 targets, HL‑1 cells were transfected with Flag‑OTUD5 or empty vector (24 h), lysed, and immunoprecipitated with anti‑Flag beads; LC–MS/MS (Mouhe Biotechnology) identified proteins enriched in the Flag‑OTUD5 group over control as candidate interactors.

### 2.13 Statistical analysis

GraphPad Prism 9.5.1 was used for all analyses. Data are shown as mean ± SD. Normality and variance homogeneity were verified prior to testing. Two‑group comparisons used unpaired two‑tailed Student's t‑tests; multi‑group comparisons used one‑way ANOVA followed by Tukey's post hoc test. Significance was set at *p* value < 0.05 (*p < 0.05, **p < 0.01, ***p < 0.001, ****p < 0.0001).

## 3 RESULTS

### 3.1 Identified OTUD5 as an upregulated regulatory deubiquitinating enzyme implicated in septic cardiomyopathy

**FIGURE 1:**
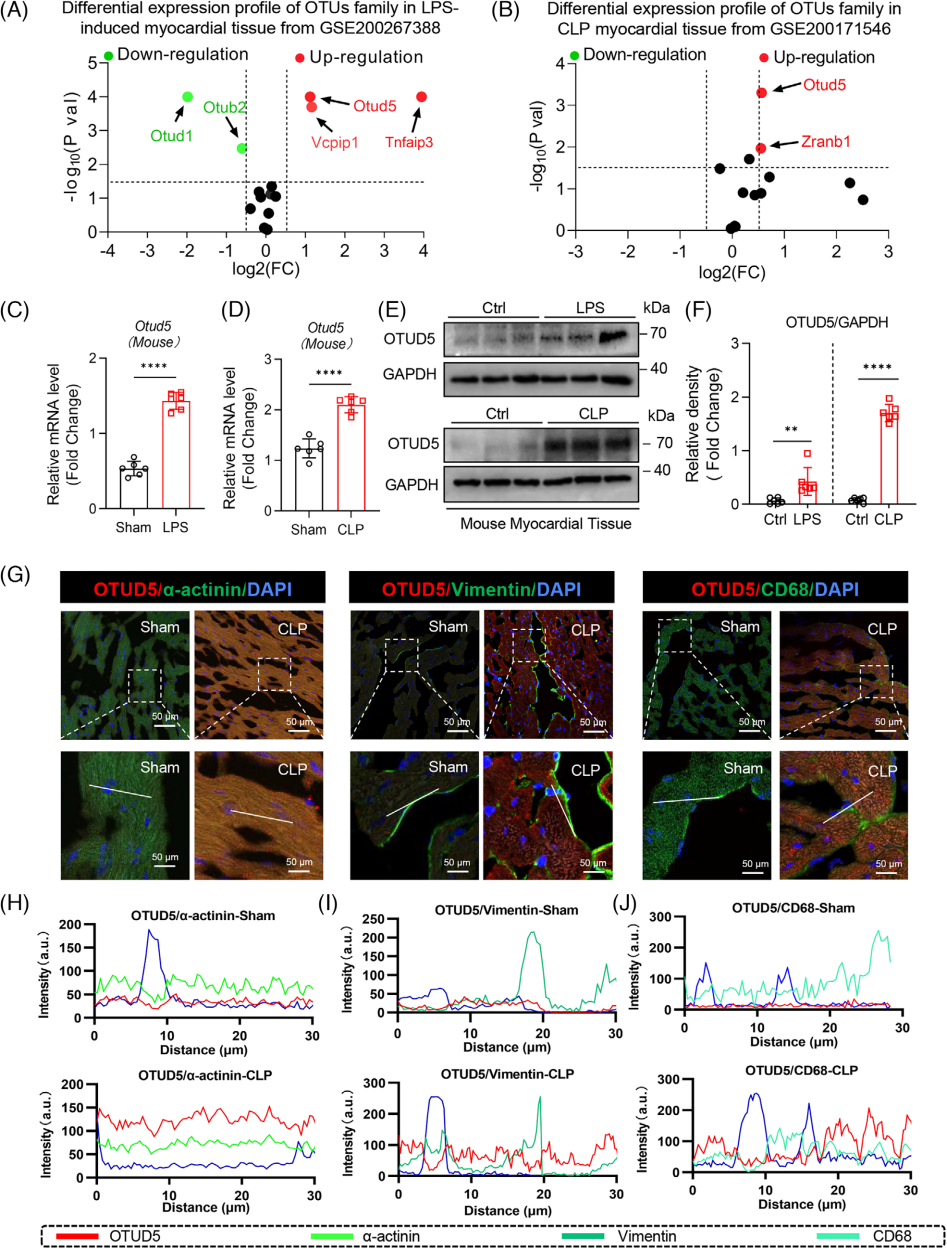
Identified OTUD5 as an upregulated regulatory deubiquitinating enzyme implicated in septic cardiomyopathy. (A and B) Transcriptome profiling of deubiquitinating enzymes (DUBs) in a murine model of septic myocardial injury based on public high‐throughput RNA sequencing. Results are presented as a volcano plot, where the *x*‐axis represents the log_2_‐transformed fold change (log_2_FC) and the *y*‐axis denotes the negative log_10_‐transformed statistical significance (−log_10_P). The plot shows differential expression of DUBs between the septic myocardial injury group and the sham‐operated group: red data points indicate significantly upregulated DUBs, green data points represent significantly downregulated DUBs, and black data points denote DUBs with no significant expression changes. The threshold for differential expression was set at |fold change| > 1.5 and *p *< .05. *n* = 3. (C and D) Real‐time quantitative PCR analysis of OTUD5 mRNA levels in murine models of septic myocardial injury induced by LPS or caecal ligation and puncture (CLP). *n* = 6. (E) Western blotting detection of OTUD5 protein expression in murine models of septic myocardial injury induced by LPS or CLP. *n* = 6. (F) Statistical analysis of OTUD5 protein levels in myocardial tissues. *n* = 6. (G–J) Representative immunofluorescence images (G) of OTUD5 (red) co‐stained with α‐actin (green, cardiomyocyte marker), vimentin (green, fibroblast marker), or CD68 (green, macrophage marker) and quantitative analysis (H–J) in heart sections from CLP and sham‐operated mice. Statistical significance was defined as: **p < .01 and ****p < .0001.

Deubiquitinases comprise seven major subfamilies. The USP family has been extensively studied in sepsis,^19^, ^20^, ^21^, ^22^ while the OTU family remains largely unexplored despite its established role in innate immune regulation. To identify key DUBs in septic cardiomyopathy (SCM), we analysed publicly available GEO datasets, specifically GSE200267388^23^ and GSE200171546,^24^ with a focus on the OTU family. Our analysis revealed that *Otud1* and *Otud2* were downregulated, whereas *Otud5*, *Vcpip1* and Tnfaip3 exhibited upregulation (Figure 1A,B). Notably, *Otud5* was the only DUB that showed significant upregulation in both LPS and CLP models. We further validated these findings using mouse SCM models induced by LPS or CLP. qRT‐PCR and western blotting confirmed that OTUD5 mRNA and protein levels were significantly elevated in myocardial tissue following modelling (Figure 1C–F). Furthermore, immunofluorescence double‐labelling assays demonstrated a specific high expression of OTUD5 in cardiomyocytes (Figure 1G, Figure S1). Meanwhile, the expression of OTUD5 was lower in primary fibroblasts and primary macrophages, while it was higher in neonatal rat primary cardiomyocytes (NRCMs), which was consistent with the results of immunofluorescence (Figure S2). Collectively, these observations implicate OTUD5 in SCM pathogenesis.

### 3.2 OTUD5 modulates pyroptosis in vitro in septic cardiomyopathy

**FIGURE 2:**
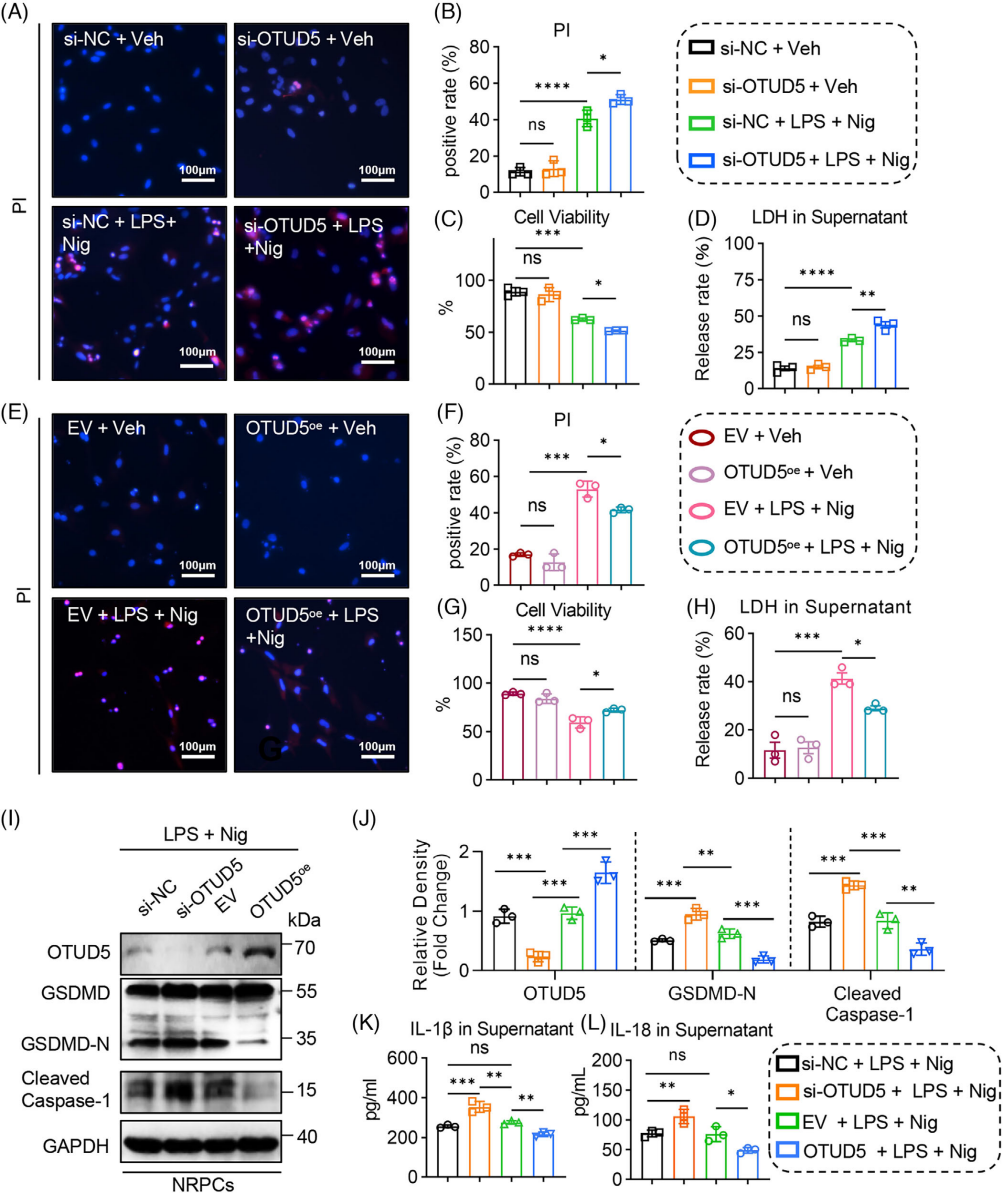
OTUD5 modulates pyroptosis in vitro in septic cardiomyopathy. Neonatal rat primary cardiomyocytes were transfected with si‐NC or si‐OTUD5, followed by serum‐ and glucose‐free medium replacement. Cells were stimulated with LPS (6 h), and Nigericin (Nig, 1 h before collection) was added for the final hour. For overexpression experiments, cells were transfected with empty vector (EV) or OTUD5 plasmid, treated with LPS (6 h), and stimulated with Nigericin (Nig, 1 h before collection). (A and B) Hoechst/PI double staining for pyroptosis detection in cardiomyocyte and corresponding statistical analysis. (C) Cell viability assessed by CCK‐8 assay. (D) LDH release in cell culture supernatants. (E and F) Hoechst/PI double staining for pyroptosis detection in cardiomyocytes and corresponding statistical analysis. (G) Cell viability assessed by CCK‐8 assay. (H) LDH release in cell culture supernatants. (I and J) Western blotting analysis of GSDMD‐N (cleaved GSDMD) and Cleaved‐Caspase‐1 expression in primary cardiomyocytes, with corresponding statistical bar graphs. (K) IL‐1β secretion in cell culture supernatants. (L) IL‐18 secretion in cell culture supernatants. Statistical significance was defined as: *p < .05, **p < .01**, ***p < .001 and ****p < .0001. The abbreviation ‘NS’ indicates no statistical significance (*p* > .05). *n* = 3.

Given the emerging significance of pyroptosis in myocardial injury,^25^, ^26^ we conducted an investigation into the role of OTUD5 using an LPS/Nig‐induced pyroptosis model^10^, ^27^, ^28^ involving NRCMs. Silencing OTUD5 significantly increased the population of cardiomyocytes that were positive for PI staining (Figure 2A,B), concomitantly resulting in reduced cell viability (Figure 2C) and elevated LDH release (Figure 2D). Conversely, the overexpression of OTUD5 resulted in a decrease in the number of PI‐positive cardiomyocytes (Figure 2E,F), an enhancement in cell viability (Figure 2G), and a reduction in LDH release (Figure 2H). Moreover, we identified that OTUD5 negatively regulates crucial markers associated with pyroptosis, particularly GSDMD‐N and Cleaved‐caspase‐1. Knocking down OTUD5 can increase their levels, while overexpression results in a decrease (Figure 2I,J). In addition, the knockdown of OTUD5 leads to an augmented release of IL‐1β and IL‐18, whereas its overexpression diminishes the IL‐1β and IL‐18 level (Figure 2K,L). Collectively, these in vitro findings underscore the regulatory function of OTUD5 in modulating pyroptosis.

### 3.3 OTUD5 interacts with the NACHT domain of NLRP3

**FIGURE 3:**
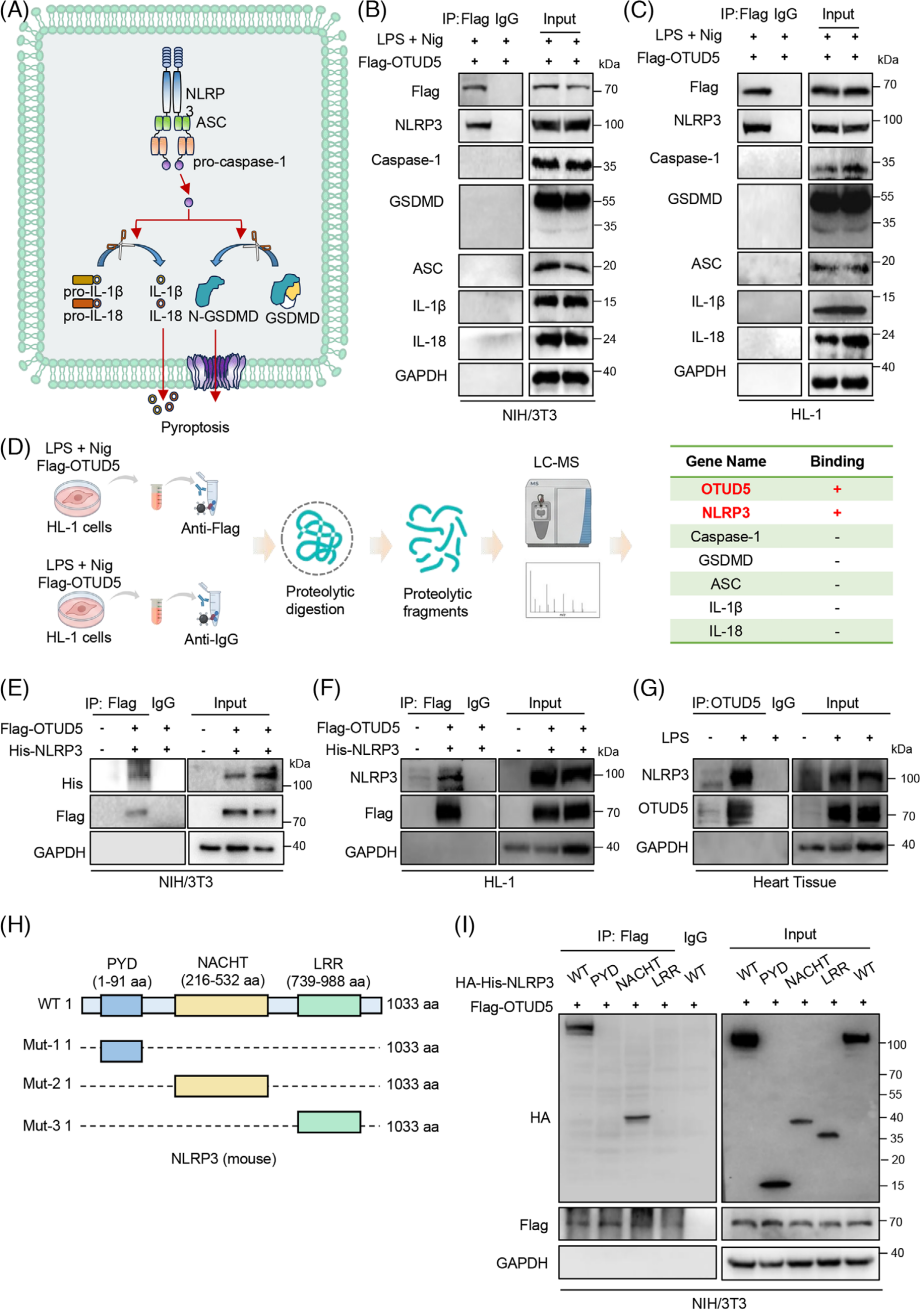
OTUD5 interacts with the NACHT domain of NLRP3. (A) Schematic diagram of pyroptosis. (B and C) NIH/3T3 and HL‐1 cells were transfected with Flag‐OTUD5 plasmid, then stimulated with LPS + Nig to establish the pyroptosis model. Cell lysates were prepared with NP40 lysis buffer, and Co‐IP was performed using anti‐Flag antibody (or IgG as control) to pull down Flag‐OTUD5‐associated complexes. (D) Workflow for screening OTUD5 interacting proteins. The Flag‐OTUD5 plasmid was transfected into mouse HL‐1 cardiomyocytes for overexpression, followed by Co‐IP using anti‐Flag magnetic beads with IgG beads as a control. The precipitated protein complexes were analysed by LC–MS/MS, with the table showing identified potential substrates of OTUD5. (E‐FF) NIH/3T3 cells (C) and HL‐1 cells (D) were co‐transfected with Flag‐OTUD5 and His‐NLRP3 plasmids to overexpress both proteins. Cell lysates were prepared using NP40 lysis buffer, and Co‐IP was performed with anti‐Flag antibody (or IgG as a negative control) to detect potential interactions involving Flag‐OTUD5. (G) Co‐IP assay in mouse myocardial tissue lysates. Protein extracts from animal hearts were prepared, and IP was performed using anti‐OTUD5 antibody (or IgG as control) to identify endogenous OTUD5‐binding partners. (H) Schematic illustration of the NLRP3 domain deletion construct. (I) Co‐IP of wt‐NLRP3, mut‐NLRP3 and OTUD5 in NIH/3T3 cells co‐transfected with overexpression plasmids of HA‐His‐wt‐NLRP3, HA‐His‐mut‐NLRP3 and Flag‐OTUD5. Exogenous normal or mutated NLRP3 was immunoprecipitated by anti‐Flag antibody.

Cardiomyocyte pyroptos is the core molecular hub that drives the pathogenesis of SCM. Upon NLRP3 inflammasome activation, Caspase‑1 is recruited and activated, which cleaves GSDMD to form membrane pores and promotes IL‑1β/IL‑18 maturation, ultimately causing cardiomyocyte death and cardiac dysfunction.^29^, ^30^ As a deubiquitinating enzyme (DUB), OTUD5 may exert its function by modulating substrate ubiquitination, thus identifying its associated substrates is essential. We have established that OTUD5 plays a role in regulating pyroptosis. Consequently, we conducted an investigation in NIH/3T3 and HL‐1 cells overexpressing Flag‐OTUD5 and stimulated with LPS/Nig to screen for potential interactions among proteins related to pyroptosis, including NLRP3, Caspase‐1, GSDMD, ASC, IL‐1β and IL‐18 using Co‐IP. Our findings reveal that OTUD5 interacts with NLRP3 (Figure 3A–C). Co‑IP/LC–MS/MS screening in OTUD5‑overexpressing cells identified NLRP3 as a candidate substrate (Figure 3D and Figure S3A,B). This interaction was further validated in NIH/3T3 cells, HL‐1 cells (Figure 3E,F). Co‐IP of endogenous proteins from mouse heart tissue confirmed the OTUD5–NLRP3 interaction (Figure 3G). It demonstrates that the OTUD5–NLRP3 interaction occurs under physiological conditions in cardiac tissue, thereby providing direct evidence for the in vivo relevance of the OTUD5–NLRP3 axis in the heart. NLRP3 comprises a central NACHT domain, a C‐terminal leucine‐rich repeat (LRR) domain, and an N‐terminal pyrin domain (PYD) effect region (Figure 3H). Co‐IP experiments using mutants of the NLRP3 domains demonstrated that OTUD5 selectively targets the NACHT region of NLRP3 (Figure 3I). These observations indicate that NLRP3 represents a plausible functional substrate through which OTUD5 mediates its regulatory role in SCM.

### 3.4 The C224 site of OTUD5 is responsible for the deubiquitination of NLRP3 to regulate its activity and alleviate pyroptosis

**FIGURE 4:**
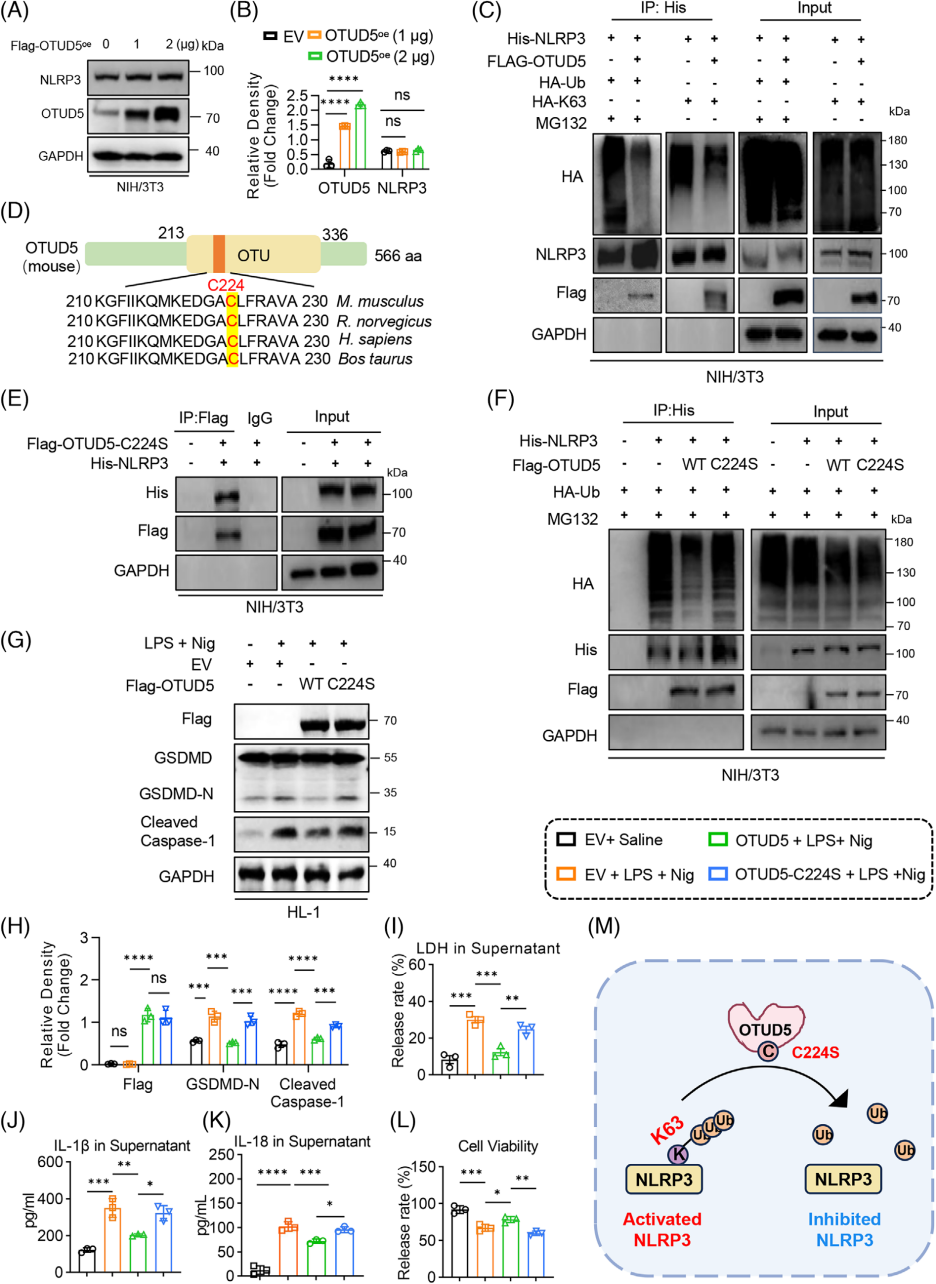
The C224 site of OTUD5 is responsible for the deubiquitination of NLRP3 to regulate its activity and alleviate pyroptosis. (A and B) Dose‐dependent overexpression of Flag‐OTUD5 in NIH/3T3 cells. Cells were transfected with 0, 1 or 2 µg of Flag‐OTUD5 plasmid, and the expression levels of Flag‐OTUD5 and NLRP3 were detected by Western blotting, with corresponding statistical analysis. *n *= 3. (C) NIH/3T3 cells were co‐transfected with His‐NLRP3, HA‐Ub (wild‐type ubiquitin), HA‐Ub‐K63 (K63‐only mutant ubiquitin), and Flag‐OTUD5 plasmids. Cells were treated with MG132 (10 µM, 6 h) to inhibit proteasomal degradation, and Co‐IP was performed to assess the abundance of HA‐Ub and HA‐Ub‐K63 conjugated to NLRP3. (D) Schematic illustration of the OTUD5‐C224 catalytic site mutation and conservation analysis showing that the C224 residue is highly conserved across species (including musculus, norvegicus, *H. sapiens* and *Bos taurus*). (E) NIH/3T3 cells were co‐transfected with His‐NLRP3, Flag‐OTUD5 and Flag‐OTUD5‐C224S plasmids. Co‐IP was used to evaluate the binding affinity between OTUD5‐C224S and NLRP3. (F) NIH/3T3 cells were co‐transfected with HA‐Ub, His‐NLRP3, Flag‐OTUD5 and Flag‐OTUD5‐C224S plasmids. Cells were treated with MG132 (10 µM, 6 h) prior to sample collection, and Co‐IP was performed to quantify HA‐Ub modification on NLRP3. (G and H) Western blotting analysis of GSDMD‐N and Cleaved Caspase‐1 protein levels, with corresponding statistical bar graphs. *n *= 3. (I) Percentage of LDH release. *n *= 3. (J) IL‐1β secretion in cell culture supernatants. *n *= 3. (K) Cell viability assessed by CCK‐8 assay. *n *= 3. (L) IL‐18 secretion in cell culture supernatants. *n* = 3. (M) Schematic mechanism illustrating how the C224 cysteine residue in OTUD5 regulates K63‐linked ubiquitination of NLRP3. Statistical significance was defined as: *p < .05, **p < .01, ***p < .001 and ****p < .0001.

To elucidate the regulatory role of OTUD5 in NLRP3 modulation, we overexpressed varying amounts of Flag‐OTUD5 in NIH/3T3 cells and observed that NLRP3 protein levels remained unchanged, suggesting that OTUD5 does not influence NLRP3 stability (Figure 4A,B). Co‐transfection experiments involving His‐NLRP3, HA‐Ub, HA‐Ub‐K63, and Flag‐OTUD5, followed by treatment with MG132, demonstrated that ubiquitination of NLRP3 occurs. Notably, overexpression of OTUD5 specifically diminished K63‐linked ubiquitination of NLRP3 (Figure 4C). We also found that in LPS‐induced and CLP‐induced myocardial tissue of OTUD5 knockout mice, the K63 ubiquitination level of NLRP3 was significantly increased (Figure S6A,B). Research indicates that the catalytic centre of OTUD5 includes cysteine 224 (C224),^31^ a residue conserved across species (Figure 4D). We subsequently generated a catalytic dead mutant designated as Flag‐OTUD5‐C224S. Co‐IP assays confirmed that the OTUD5‐C224S mutant retains binding to NLRP3 (Figure 4E). However, it was found to lack the capacity to deubiquitinate NLRP3 when compared to its wild‐type counterpart (Figure 4F). This finding highlights the critical importance of the C224 site for OTUD5‐mediated deubiquitination of K63 chains on NLRP3 and subsequently inhibiting activation of the inflammasome.

In contrast to wild‐type OTUD5, the C224S mutant did not effectively reduce LPS/Nig‐induced levels of GSDMD‐N or Cleaved Caspase‐1 (Figure 4G,H), did not inhibit suppress LDH, IL‐1β and IL‐18 release (Figure 4I–K), and lost its protective effect on cardiomyocyte viability (Figure 4L). These data collectively demonstrate that cysteine residue C224 plays an essential role in mediating both deubiquitination processes affecting NLRP3 and inhibition thereof regarding its activation (Figure 4M).

### 3.5 Cardiomyocyte‐specific knockout of OTUD5 exacerbates CLP‐induced septic myocardial injury and pyroptosis

**FIGURE 5:**
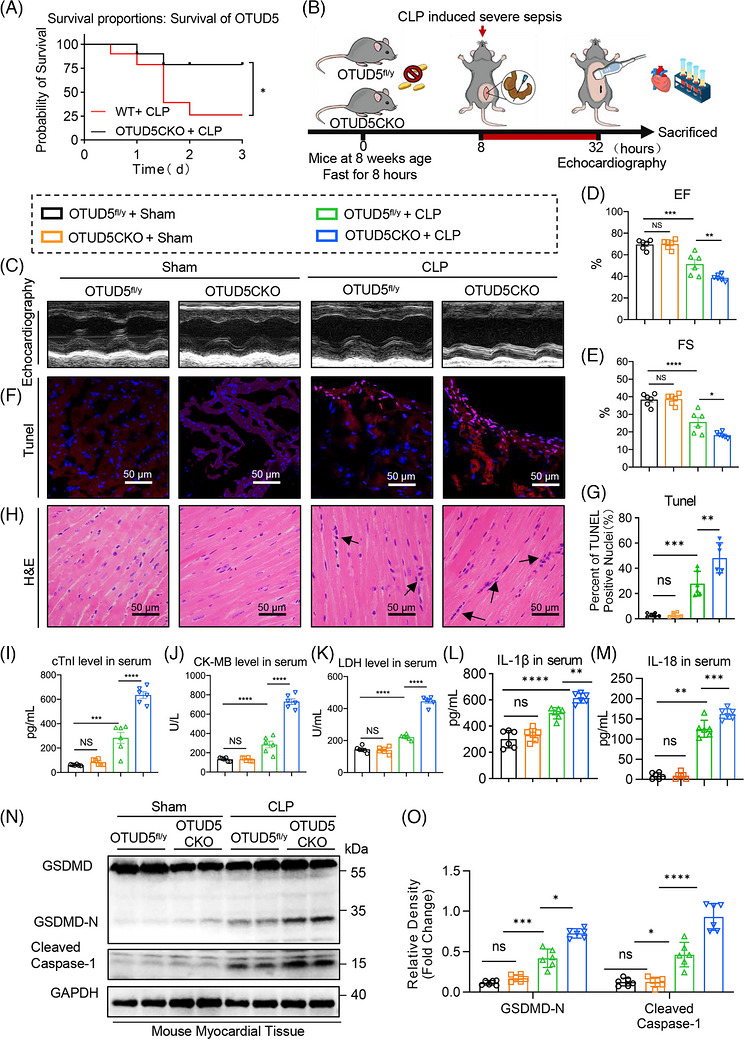
Cardiomyocyte‐specific knockout of OTUD5 aggravates CLP‐induced septic myocardial injury and pyroptosis. (A) Survival curves of WT mice and OTUD5CKO mice following caecal ligation and puncture (CLP) surgery to induce septic cardiomyopathy (observation period: 72 h). *n* = 10. (B) Experimental workflow for the CLP‐induced septic cardiomyopathy mouse model. (C) M‐mode echocardiography images of mouse hearts from each group. (D) Quantitative analysis of EF %. *n* = 6. (E) Quantitative analysis of FS %. n = 6. (F and G) TUNEL staining to detect cardiomyocyte pyroptosis (apoptotic nuclei labelled with red fluorescence), with corresponding statistical analysis of pyroptotic cell proportion. *n* = 6. (H) Representative H&E‐stained images of myocardial tissue from each group. (I) Serum cTnT levels from each group in mice. *n* = 6. (J) Serum CK‐MB activity from each group in mice. *n* = 6. (K) Serum LDH release from each group in mice. *n *= 6. (L) Myocardial tissue supernatant IL‐1β content. *n* = 6. (M) Myocardial tissue supernatant IL‐18 content. *n* = 6. (N and O) Western blotting analysis of GSDMD‐N and Cleaved Caspase‐1 protein levels in mouse myocardium, with corresponding statistical bar graphs. *n* = 6. Statistical significance was defined as: *p < .05, **p < .01, ***p < .001 and ****p < .0001. The abbreviation ‘NS’ indicates no statistical significance (*p* > .05).

To investigate the in vivo function of OTUD5, we generated cardiomyocyte‐specific OTUD5 knockout mice (OTUD5‐CKO) on a C57BL/6J background (Figure S4A–D; Table S2). In the CLP‐induced survival model, OTUD5‐CKO mice exhibited significantly diminished survival rates compared to the control group (Figure 5A). Assessment of cardiac function conducted 24 h post‐CLP (Figure 5B) indicated marked ventricular systolic dysfunction in CLP‐induced OTUD5‐CKO mice, characterized by reductions in both EF% and FS% (Figure 5C–E; Table S3). TUNEL staining (Figure 5F,G) and H&E staining (Figure 5H) further validated the exacerbated myocardial pathological damage observed in CLP‐induced OTUD5‐CKO mice. Also, LDH, CK‐MB, and cTnI in serum were all notably increased in OTUD5‐CKO mice under LPS treatment (Figure 5I–K). In addition, serum IL‐1β and IL‐18 (Figure 5L,M), along with GSDMD‐N and Cleaved‐caspase‐1 concentrations were altered in OTUD5‐CKO mice subjected to CLP (Figure 5N,O). These findings indicate that OTUD5 plays a protective role against CLP‐induced septic myocardial injury by modulating the Caspase‐1/GSDMD‐mediated pyroptosis pathway.

### 3.6 Cardiomyocyte‐specific knockout of OTUD5 exacerbates LPS‐induced septic myocardial injury and pyroptosis

The survival rates of OTUD5‐CKO mice following LPS challenge (10 mg/kg, i.p.) were significantly diminished (Figure S5A). Cardiac function, assessed via echocardiography (Figure S5B), revealed that OTUD5‐CKO mice exhibited more severe ventricular systolic dysfunction post‐LPS compared to their OTUD5^fl/y^ controls, characterized by significantly reduced EF% and FS% (Figure S5C–E and Table S4). TUNEL‑positive cell counts were significantly higher in OTUD5‑CKO hearts, indicating enhanced cell death (Figure S5F,G). H&E staining demonstrated disordered myocardial fibres, interstitial oedema and inflammatory cell infiltration in these knockout animals (Figure S5H). Moreover, serum levels of cardiac injury markers LDH, CK‐MB and cTnI were markedly elevated in the OTUD5‐CKO mice subjected to LPS (Figure S5I–K). In addition, mouse serum IL‐1β and IL‐18 were significant higher (Figure S5L,M), and elevated GSDMD‑N/cleaved Caspase‑1 protein levels in LPS‐induced OTUD5‐CKO hearts (Figure S5N,O). These results highlight that OTUD5 confers significant protection against pyroptosis in both LPS and CLP models.

### 3.7 OTUD5 ameliorates CLP‐induced septic myocardial injury dependent on NLRP3

**FIGURE 6:**
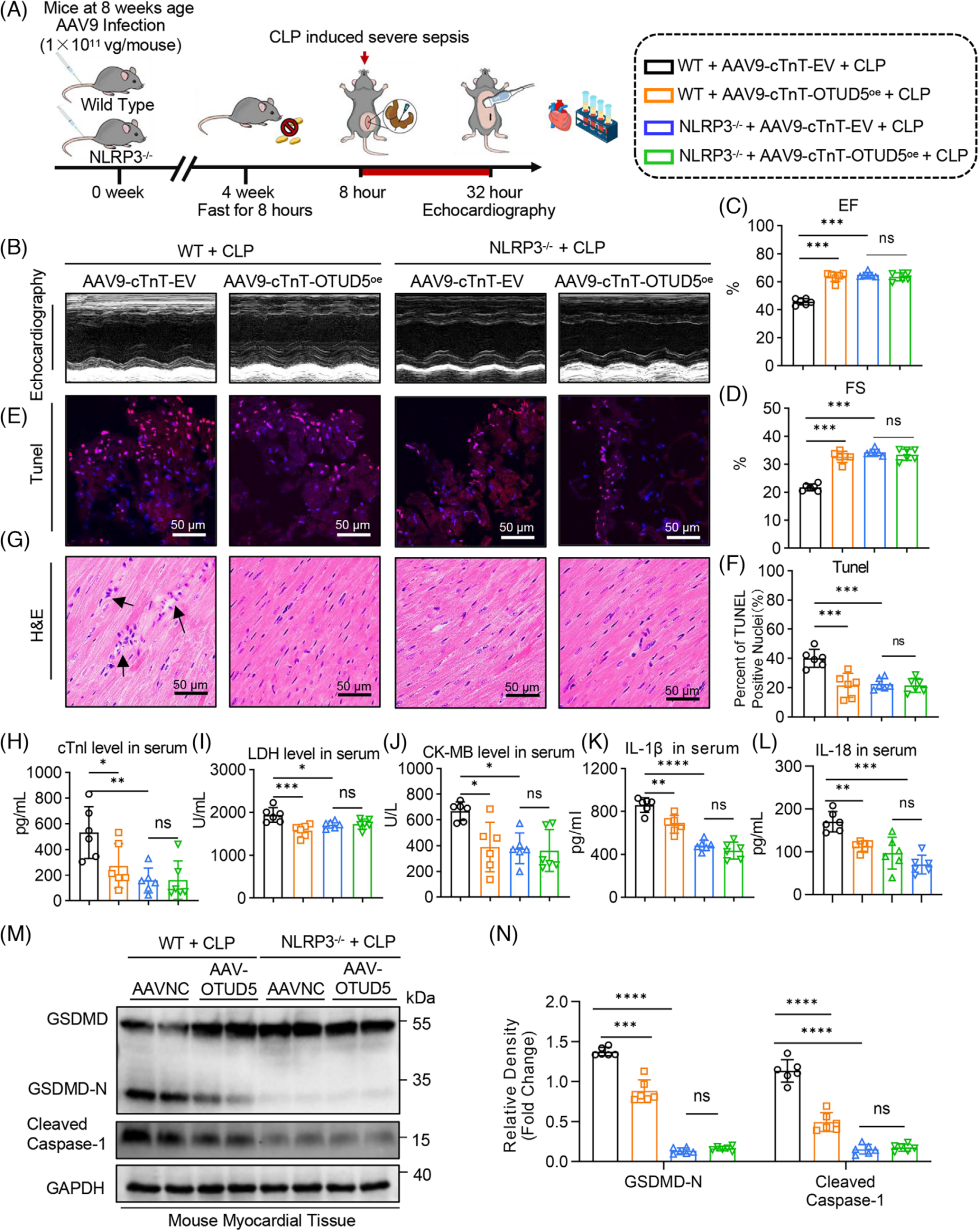
OTUD5 ameliorates CLP‐induced septic myocardial injury dependent on NLRP3. Eight‐week‐old wild‐type (WT) and NLRP3‐knockout (NLRP3^−/−^) mice were injected via the tail vein with either AAV9‐cTnT‐OTUD5oe (OTUD5‐overexpressing vector) or corresponding AAV9‐cTnT‐EV (empty vector control) at a dose of 1 × 10^11^ viral genomes per mouse. Following a 4‐week transduction period, mice were fasted and subjected to caecal ligation and puncture (CLP) surgery to induce polymicrobial sepsis. Cardiac function was evaluated by echocardiography at 24 h post‐CLP, after which heart tissues and serum samples were collected for subsequent analysis. (A) Schematic illustration of the experimental procedure for CLP‐induced septic cardiomyopathy with AAV9‐based intervention. (B) M‐mode echocardiography images of mouse hearts from each experimental group. (C) Quantitative analysis of EF %. *n* = 6. (D) Statistical analysis of FS %. *n* = 6. (E and F) TUNEL staining to detect cardiomyocyte pyroptosis (apoptotic nuclei labelled with red fluorescence), with corresponding statistical bar charts showing the proportion of pyroptotic cells. *n* = 6. (G) Representative H&E‐stained images of myocardial tissue from each group. (H) Impact of OTUD5 deficiency on serum cardiac cTnT levels in CLP‐induced septic cardiomyopathy. *n* = 6. (I) Effect of OTUD5 deficiency on serum CK‐MB release in CLP‐challenged mice. *n* = 6. (J) Influence of OTUD5 deficiency on serum LDH release in the CLP model. *n* = 6. (K) Alterations in myocardial tissue supernatant IL‐1β content due to OTUD5 deficiency in CLP‐induced injury. *n* = 6. (L) Alterations in myocardial tissue supernatant IL‐18 content due to OTUD5 deficiency in CLP‐induced injury. *n* = 6. (M and N) Western blotting analysis of GSDMD‐N and Cleaved Caspase‐1 protein expression in mouse myocardium, with corresponding statistical bar graphs. *n* = 6. Statistical significance was defined as: *p < .05, **p < .01, ***p < .001 and ****p < .0001. The abbreviation ‘NS’ indicates no statistical significance (*p* > .05).

To evaluate the dependence of OTUD5's cardioprotective effects on NLRP3 and assess the therapeutic potential of AAV9‐OTUD5, we used wild‐type (WT) and NLRP3‐deficient (NLRP3^−/−^) mice (Figure S7A–D). The mice underwent tail vein injections with either AAV9‐cTnT‐OTUD5 or an empty vector (EV) control (Figure S8A–D). Four weeks post‐injection, CLP surgery was performed (Figure 6A). Echocardiographic analysis revealed that treatment with the OTUD5 virus significantly improved EF and FS in WT mice following CLP. However, this improvement was notably absent in NLRP3^−/−^ mice receiving identical treatment (Figure 6B–D; Table S5). In addition, OTUD5 markedly reduced the count of cells positive for TUNEL staining in hearts of WT mice, an effect that was completely negated in NLRP3^−/−^ mice (Figure 6E,F). H&E staining further confirmed that viral therapy mitigated CLP‐induced myocardial damage in WT but not in NLRP3^−/−^ mice (Figure 6G), indicating that OTUD5's cardioprotection is dependent on NLRP3. Serum markers indicative of cardiac injury such as LDH, CK‐MB and cTnI were significantly diminished by OTUD5 administration in WT animals but showed no reduction in NLRP3^−/−^ counterparts (Figure 6H–J). Similarly, serum levels of IL‐1β and IL‐18 (Figure 6K,L), along with protein expression levels of GSDMD‐N and Cleaved‐caspase‐1 (Figure 6 M,N), exhibited a comparable trend. The loss of protective effects observed in NLRP3‐deficient mice underscores the critical role of targeting NLRP3 for OTUD5 to exert its anti‐pyroptotic actions within cardiac tissue.

## 4 DISCUSSION

This study identifies the deubiquitinating enzyme OTUD5 as being significantly upregulated in myocardial tissue subjected to sepsis induced by LPS and CLP. Cardiomyocyte‐specific knockout of OTUD5 increased severity of sepsis‑induced myocardial injury. Mechanistically, OTUD5 interacts directly with NLRP3 and used its catalytic C224 site to selectively remove K63‑ubiquitin adducts from NLRP3, thus inhibiting the activation of the NLRP3 inflammasome. Lastly, we validated the functional significance of the OTUD5‐NLRP3 axis in the CLP‑induced mouse model of SCM.

The findings of this study have identified the deubiquitinating enzyme OTUD5 as a critical endogenous protective factor in septic cardiomyopathy, offering novel insights and highly promising targets for the development of precision therapies centred on DUBs. Traditionally, treatments for septic cardiomyopathy have primarily concentrated on macroscopic hemodynamic support^32^ or broad‐spectrum anti‐inflammatory strategies,^33^ often lacking interventions that directly address the specific injury mechanisms affecting cardiomyocytes. This study confirmed that OTUD5 is notably expressed in cardiomyocytes and directly interacts with the key protein NLRP3 within the pyroptosis core pathway through its deubiquitinating enzyme activity. It selectively removes the polyubiquitination chain linked to K63, thereby inhibiting excessive activation of the NLRP3 inflammasome and subsequent pyroptosis processes. This targeted intervention approach is particularly specific and precise, mitigating potential risks of immune deficiency associated with complete inhibition of NLRP3. Moreover, when compared to other deubiquitinating enzymes such as USP7,^34^ USP13,^35^ USP20^22^ and YOD1^36^ studied in relation to septic cardiomyopathy, OTUD5 exhibits distinct advantages regarding target specificity. Firstly, OTUD5 demonstrates higher targeting specificity than its counterparts. While USP7 and USP13 influence inflammation indirectly through complex signalling networks involving transcription factors and microRNAs, this study has established that OTUD5 can bind directly to and deubiquitinate the NACHT domain of NLRP3. Such a direct mechanism enhances clarity in action pathways while reducing off‐target effects. Secondly, the mechanism of action of OTUD5 demonstrates a higher degree of precision. While USP20 similarly removes K63‐linked ubiquitin chains from NLRP3 (at K243, via its catalytic C154) and inhibits the NLRP3‐ASC interaction to suppress pyroptosis, its expression is markedly downregulated in the septic heart, representing a passive loss of protection; conversely, OTUD5 is endogenously upregulated in both LPS and CLP models, signifying an actively mobilized host defence mechanism. More critically, our data establish that OTUD5 selectively cleaves K63‐linked polyubiquitin chains from the NACHT domain of NLRP3. This anti‐pyroptotic function is strictly dependent on the catalytic cysteine C224. Importantly, this activity does not affect total NLRP3 protein stability. This feature fundamentally distinguishes OTUD5 from YOD1, which cleaves K48‑conjugated ubiquitin moieties, thereby stabilizing NLRP3 and paradoxically aggravating pyroptosis and cardiac injury. OTUD5 therefore functions as a pure activity brake that inhibits inflammasome activation without causing the deleterious NLRP3 accumulation that defines the pathogenic role of YOD1. In addition, this mechanism concurrently avoids the immunosuppressive risks associated with complete NLRP3 ablation. This approach reflects an ‘active immobilization’ rather than a ‘protein clearance’ paradigm, thereby curbing excessive activation of NLRP3 while retaining its potential physiological functions to the greatest extent possible and alleviating concerns regarding immune deficiency that could arise from complete inhibition.

Of paramount importance, this study successfully demonstrated specific overexpression of OTUD5 in cardiomyocytes in vivo using an AAV9 viral vector, leading to significant amelioration of myocardial injury and dysfunction induced by the CLP model. This finding directly validates the feasibility of employing gene therapy strategies targeting OTUD5. Looking forward, OTUD5 emerges as a highly potential therapeutic target itself. The development of small molecule agonists or gene delivery systems akin to AAV9 aimed at enhancing either the activity or expression levels of myocardial OTUD5 may evolve into an innovative etiological therapeutic strategy targeting programmed cell death mechanisms within cardiomyocytes during septic cardiomyopathy. Thus, opening new horizons for improving cardiac prognosis in patients suffering from sepsis.

There is a broad consensus that the NLRP3 inflammasome represents a key intervention point. Its inhibition or modulation holds therapeutic promise for both sepsis and the cardiac damage frequently associated with it.^29^, ^37^ Targeting NLRP3 or its regulatory pathways has shown promise in SCM models.^8^, ^9^, ^26^ However, direct pharmacological targeting of NLRP3 presents significant challenges due to the absence of a defined drug‐binding pocket,^38^ an unresolved complete structure, potential immunosuppression associated with systemic inhibition,^39^ and off‐target effects from existing inhibitors such as MCC950,^40^ OLT1177^41^ and Tranilast.^42^ Consequently, direct pharmacological targeting of NLRP3 presents significant hurdles. As a result, focusing on specific regulatory pathways associated with NLRP3 has emerged as an important alternative strategy. The notable contribution of this study lies not only in confirming that ‘inhibiting NLRP3 is beneficial’ but also in uncovering the role of OTUD5 as an upstream regulatory mechanism. Rescue experiments conducted using NLRP3 gene knockout mice clearly demonstrated that the cardioprotective effect of OTUD5 is entirely contingent upon the presence of NLRP3. This finding not only validates OTUD5 as a target but also suggests that future therapeutic approaches should prioritize ‘regulating’ rather than merely ‘abolishing’ NLRP3 activity. Therefore, this study advances our understanding for future treatments of septic cardiomyopathy by proposing a novel therapeutic paradigm that transcends direct inhibition of NLRP3. This approach emphasizes safety and precision: specifically targeting its upstream regulatory factors to effectively ‘precisely brake’ the NLRP3 inflammasome and striking a better balance between efficacy and safety.

There have been no reports on small molecule agonists or activators targeting OTUD5. Currently, the strategies for enhancing the function of OTUD5 are entirely dependent on AAV‐mediated gene overexpression. And this method has demonstrated significant preclinical efficacy in various disease models, including diabetic nephropathy,^31^ myocardial ischemia/reperfusion injury,^16^ acute kidney injury models,^43^ and our current findings in cardiomyopathy. In this study, we have confirmed that the protective effect of OTUD5 depends on its cysteine C224 active site and can specifically remove K63‐linked ubiquitin chains in STAT3 without affecting the stability of the STAT3 protein. Moreover, the catalytic site of C224 and the adjacent substrate binding groove in the structure are all drug designable, and can be used to construct allosteric small molecule agonists or molecular adhesives to enhance the endogenous activity of OTUD5 and improve pathological myocardial remodelling. Cardiovascular diseases remain the leading cause of death worldwide, yet lifestyle modification and risk factor control can effectively reduce their incidence and mortality.^44^

Nevertheless, this research has certain limitations. The specific deubiquitination sites on NLRP3 that are targeted by OTUD5 remain undetermined. Future studies using mass spectrometry may identify these sites for precise drug targeting. In addition, the downstream consequences of OTUD5‐mediated NLRP3 deubiquitination in relation to interactions with other inflammasome components warrant further investigation. Our findings are primarily derived from rodent models and cell lines; potential interspecies differences call for validation through clinical samples to assess their translational relevance. And the unavailability of myocardial tissue from septic patients for direct OTUD5 expression–prognosis correlation, as endomyocardial biopsy is ethically precluded in this critically ill cohort, and postmortem specimens are confounded by protein degradation that would mask the dynamic ubiquitination changes central to our mechanistic findings.

In conclusion, this study demonstrates that OTUD5 mitigates SCM by deubiquitinating NLRP3. This process specifically involves the reduction of K63‐linked ubiquitination, thereby inhibiting inflammasome activation and subsequent pyroptosis in cardiomyocytes. These findings strengthen our understanding of the mechanisms through which OTUD5 operates in SCM and we identify it as a promising novel therapeutic target.

## AUTHOR CONTRIBUTIONS

*Conceptualization*: Guang Liang and Bozhi Ye. *Methodology*: Yucheng Jiang, Zhihan Jia and Zhaozheng Zheng. *Investigation*: Yucheng Jiang, Zhihan Jia, Zhaozheng Zheng and Qingqing Zhao. *Writing—original draft*: Yucheng Jiang. *Writing—review and editing*: Naijin Zhang, Guang Liang and Bozhi Ye. *Funding acquisition*: Naijin Zhang, Guang Liang and Bozhi Ye.

## CONFLICT OF INTEREST STATEMENT

The authors declare no conflicts of interest.

## ETHICS STATEMENT

All animal procedures were approved by the Animal Experiment Ethics Committee of Wenzhou Medical University (Ethical Approval No.: wydw2024‐0164).

## Supporting information



Supporting Information

## Data Availability

The data supporting the findings of this study are available from the corresponding author upon reasonable request.
