# Supplementary material for: Cardiomyocyte‐enriched OTUD5 alleviates septic cardiomyopathy by promoting NLRP3 deubiquitination and inhibiting NLRP3 inflammasome activation
Source: Clin Transl Med. 2026 Jul 12;16(7):e70743. doi: 10.1002/ctm2.70743 (PMC13357682; doi:10.1002/ctm2.70743)
Supplement: Supplementary file 1 — Supporting Information [file CTM2-16-e70743-s001.docx]

***Supplementary Files***

**Cardiomyocyte-Enriched OTUD5 Alleviates Septic Cardiomyopathy by Promoting NLRP3 Deubiquitination and Inhibiting NLRP3 Inflammasome Activation**

***Supplementary File***

Supplementary 5 Tables, 8 Figures and Legends

**Supplementary Table S1**

| Gene | Species | Pre-primer | Post-primer |
| --- | --- | --- | --- |
| *Actb* | Mouse | CCGTGAAAAGATGACCCAGA | TACGACCAGAGGCATACAG |
| *Otud5* | Mouse | AAGTCTCCCTGTGTGTTTCTTGTCG | ACTCAGAAGGCAGAGGCAGAAGG |

**Supplementary Table S2**

Mouse gene heart-specific knockout identification primers

| Primer number | Primer name | Primer sequence(5’→3‘) | Product size | remark |
| --- | --- | --- | --- | --- |
| 1 | JS00028-Otud5-5wt-tF1A | GTGACCACCTCACCAGTATAGGCT | WT: 327bp | Identification of Otud5 with X inheritance |
|  | JS00028-Otud5-5wt-tR1A | GCCAGAAGCAGACAGCTTGACCT | Fl: 405bp |  |
| 2 | T004713-F1 | CCTGCTGTCCATTCCTTATTCCATA | WT:0bp  Targeted:329bp | Identification of myh6-Cre |
|  | T004713-R1 | ATATCCCCTTGTTCCCTTTCTGC |  |  |
| 3 | H11-wt-tF1 | CAGCAAAACCTGGCTGTGGATC | WT: 412bp |  |
|  | H11-wt-tR1 | ATGAGCCACCATGTGGGTGTC |  |  |

**Supplementary Table S3**

Echocardiographic parameters in CLP-challenged mouse experiment

|  | Sham | | CLP | |
| --- | --- | --- | --- | --- |
|  | OTUD5^fl/y^ | OTUD5CKO | OTUD5^fl/y^ | OTUD5CKO |
|  | n=6 | n=6 | n=6 | n=6 |
| LVID;d, (mm) | 3.36±0.16 | 3.45±0.44^ns^ | 3.12±0.23^ns^ | 3.4±0.26^NS^ |
| LVID;s, (mm) | 2.08±0.18 | 2.13±0.35^ns^ | 2.32±0.16^ns^ | 2.78±0.16^#^ |
| LVAW;d, (mm) | 0.81±0.20 | 0.74±0.13^ns^ | 0.80±0.06^ns^ | 0.73±0.08^NS^ |
| LVAW;s, (mm) | 1.30±0.03 | 1.26±0.12^ns^ | 1.08±0.14* | 0.89±0.11^#^ |
| IVS;d, (mm) | 0.90±0.08 | 0.92±0.14^ns^ | 0.74±0.08^ns^ | 0.76±0.12^NS^ |
| IVS;s, (mm) | 1.11±0.10 | 1.17±0.14^ns^ | 0.93±0.09^ns^ | 0.85±0.13^NS^ |
| CO, (mL/min) | 14.04±2.23 | 13.48±2.42^ns^ | 10.10±1.05* | 5.22±1.81^##^ |

LVID, left ventricular internal diameter; LVAW, left ventricular anterior wall; IVS, interventricular septum; CO, cardiac output; d, diastole; s, systole. ns, represents p>0.05 vs OTUD5^fl/y^ + Sham; *, p<0.05 vs OTUD5^fl/y^ + Sham; NS, represents p>0.05 vs OTUD5^fl/y^ + CLP; #, p<0.05 vs OTUD5^fl/y^ + CLP;##, p<0.01 vs OTUD5^fl/y^ + CLP.

**Supplementary Table S4**

Echocardiographic parameters in LPS-challenged mouse experiment

|  | Saline | | LPS | |
| --- | --- | --- | --- | --- |
|  | OTUD5^fl/y^ | OTUD5CKO | OTUD5^fl/y^ | OTUD5CKO |
|  | n=6 | n=6 | n=6 | n=6 |
| LVID;d, (mm) | 3.39±0.49 | 3.28±0.23^ns^ | 3.29±0.31^ns^ | 3.73±0.36^NS^ |
| LVID;s, (mm) | 2.16±0.40 | 2.09±0.20^ns^ | 2.46±0.25^ns^ | 3.15±0.40^##^ |
| LVAW;d, (mm) | 0.76±0.10 | 0.76±0.08^ns^ | 0.82±0.15^ns^ | 0.70±0.11^NS^ |
| LVAW;s, (mm) | 1.16±0.14 | 1.13±0.11^ns^ | 1.14±0.10^ns^ | 0.93±0.14^#^ |
| IVS;d, (mm) | 0.78±0.11 | 0.84±0.07^ns^ | 0.97±0.32^ns^ | 0.87±0.21^NS^ |
| IVS;s, (mm) | 1.13±0.17 | 1.19±0.09^ns^ | 1.36±0.23^ns^ | 0.93±0.11^###^ |
| CO, (mL/min) | 16.19±1.62 | 15.23±1.63^ns^ | 9.36±1.07*** | 6.77±1.02^#^ |

LVID, left ventricular internal diameter; LVAW, left ventricular anterior wall; IVS, interventricular septum; CO, cardiac output; d, diastole; s, systole. ns, represents p>0.05 vs OTUD5^fl/y^ + Saline; ***, p<0.001 vs OTUD5^fl/y^ + Saline; NS, represents p>0.05 vs OTUD5^fl/y^ + LPS; #, p<0.05 vs OTUD5^fl/y^ + LPS; ##, p<0.01 vs OTUD5^fl/y^ + LPS; ###, p<0.001 vs OTUD5^fl/y^ + LPS.

**Supplementary Table S5**

Echocardiographic parameters of CLP -challenged and injected with AAV-OTUD5 mouse experiment

|  | AAV-NC + CLP | | AAV-OTUD5 + CLP | |
| --- | --- | --- | --- | --- |
|  | WT | NLRP3^-/-^ | WT | NLRP3^-/-^ |
|  | n=6 | n=6 | n=6 | n=6 |
| LVID;d, (mm) | 2.84±0.50 | 3.00±0.28^ns^ | 2.45±0.71^ns^ | 3.06±0.35^NS^ |
| LVID;s, (mm) | 2.23±0.39 | 1.97±0.20^ns^ | 1.66±0.50^ns^ | 2.04±0.26^NS^ |
| LVAW;d, (mm) | 0.93±0.17 | 0.70±0.11^ns^ | 0.90±0.19^ns^ | 0.73±0.17^NS^ |
| LVAW;s, (mm) | 1.16±0.22 | 1.05±0.16^ns^ | 1.25±0.11^ns^ | 1.12±0.19^NS^ |
| IVS;d, (mm) | 1.07±0.30 | 0.97±0.10^ns^ | 1.15±0.23^ns^ | 0.94±0.07^NS^ |
| IVS;s, (mm) | 1.11±0.23 | 1.15±0.05^ns^ | 1.32±0.18^ns^ | 1.11±0.08^NS^ |
| CO, (mL/min) | 5.58±2.73 | 9.06±2.00^ns^ | 5.77±3.96^ns^ | 8.44±2.73^NS^ |

LVID, left ventricular internal diameter; LVAW, left ventricular anterior wall; IVS, interventricular septum; CO, cardiac output; d, diastole; s, systole. ns, represents p>0.05 vs WT + AAV-NC + CLP; NS, represents p>0.05 vs WT + AAV-OTUD5 + CLP.

**Supplementary Figure S1
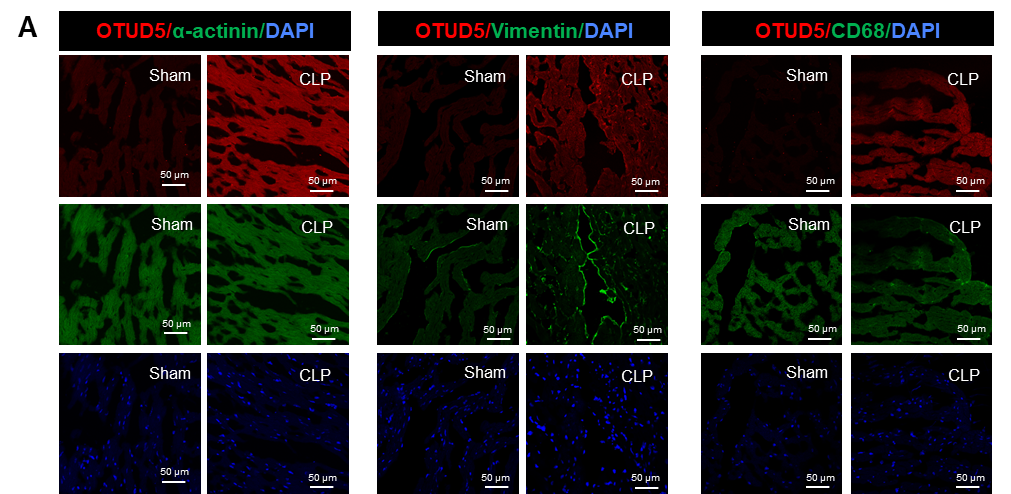
**

**Supplementary Figure S1.** **immunofluorescence double-labeling assays demonstrated a specific high expression of OTUD5 in cardiomyocytes. (A)** Representative immunofluorescence images of OTUD5 (red) co-stained with α-actin (green, cardiomyocyte marker), vimentin (green, fibroblast marker), or CD68 (green, macrophage marker) in heart sections from CLP and sham-operated mice.

**Supplementary Figure S2**

**
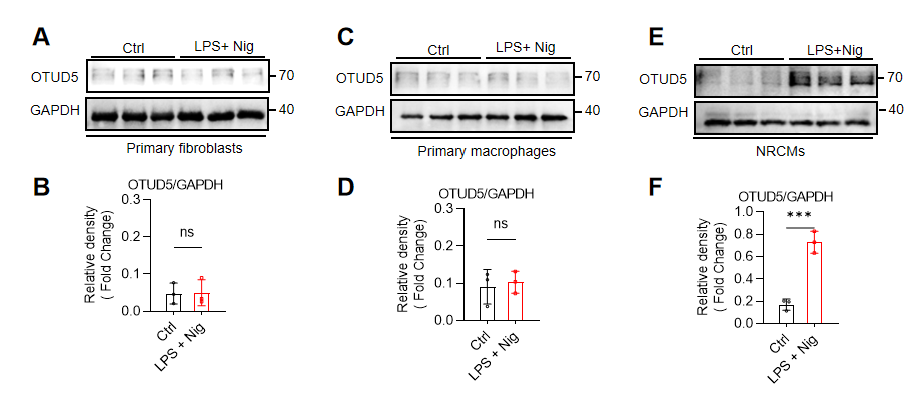
**

**Supplementary Figure S2.** **The expression of OTUD5 was lower in primary fibroblasts and primary macrophages, higher in NRCMs (A, C, E)****.** Western blotting detection of OTUD5 protein expression in primary fibroblasts, primary macrophages and NRCMs.

**(B, D, F)** Statistical analysis of OTUD5 protein levels in primary fibroblasts and primary macrophages. n= 3.

**Supplementary Figure S3**

**B**


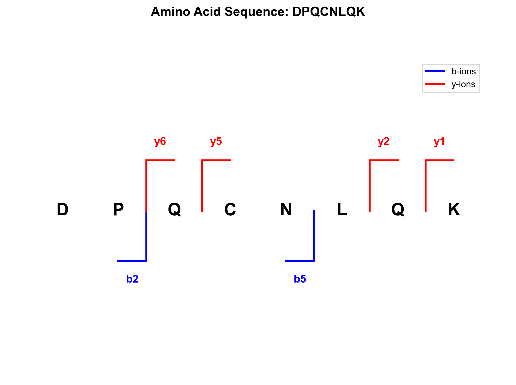

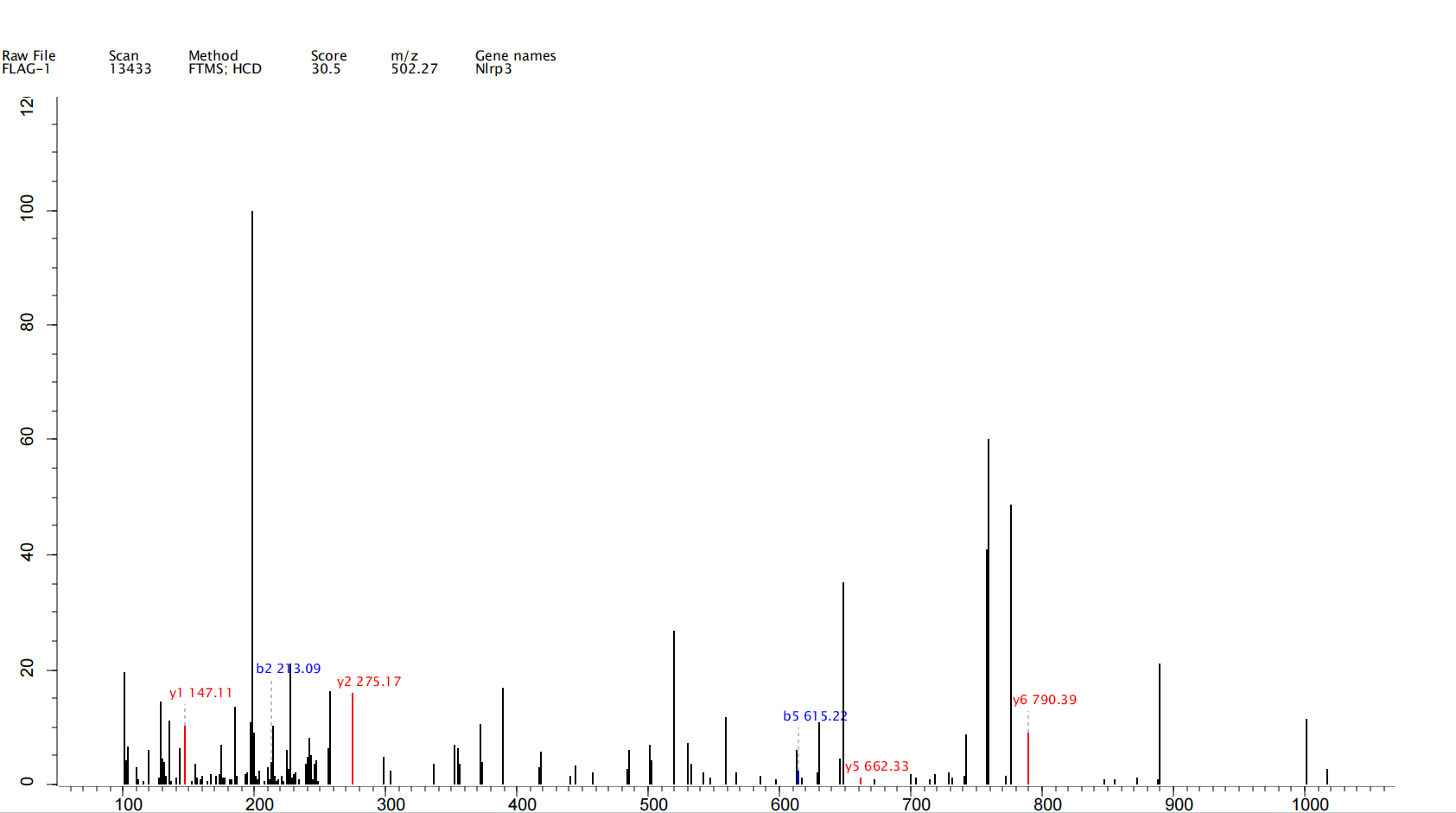


**A**

**Supplementary Figure S3. In OTUD5-overexpressed cells, we found NLRP3 as a potential substrate of OTUD5 using Co-IP and LC–MS/MS assays. (A-B)** LC-MS/MS analysis of OTUD5 binding to NLRP3 peptide segments.

**Supplementary Figure S4**

**
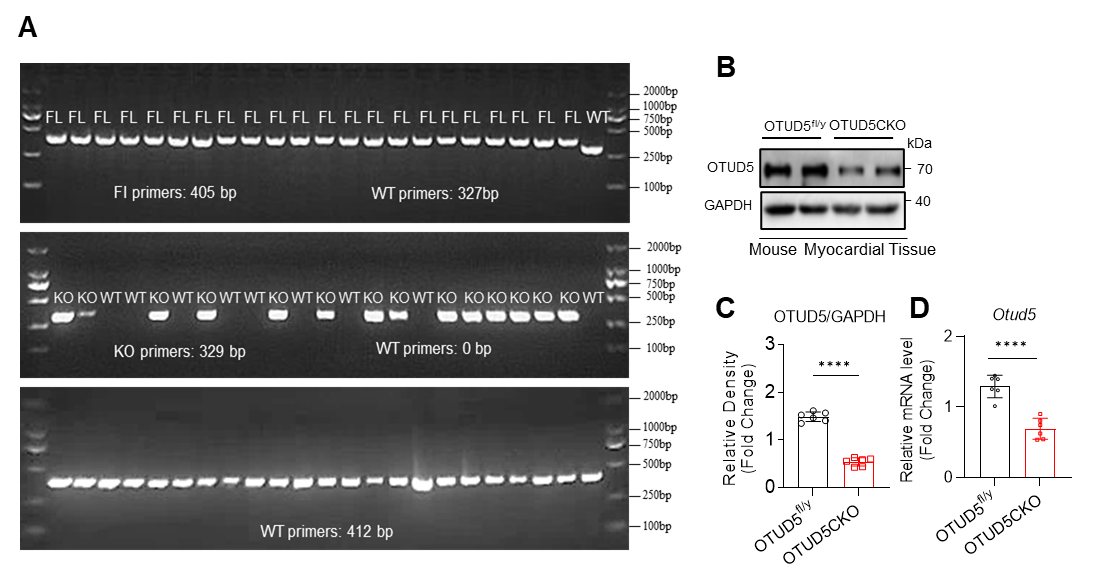
**

**Supplementary Figure S4.** **Generation and validation of cardiomyocyte-specific OTUD5 knockout mice.**

**(A)** Genotyping of mouse tail DNA to verify the specificity of OTUD5 knockout using primers 1, 2, and 3. Primer 1(JS00028-Otud5-5wt-tF1A and JS00028-Otud5-5wt-tR1A) is used to detect the floxed OTUD5 allele (X-linked inheritance), while primers 2(T004713-F1 and T004713-R1) and 3(H11-wt-tF1 and H11-wt-tR1) are used to detect the Myh6-Cre transgene. OTUD5^fl/y^ mice exhibit only the bands corresponding to primers 1 (405 bp) and 3 (412 bp). OTUD5CKO mice display bands for primers 1 (405 bp), 2 (329 bp), and 3 (412 bp).

**(B–C)** Western blot analysis of OTUD5 protein expression in myocardial tissues of OTUD5^fl/y^ and OTUD5CKO mice, along with a corresponding bar graph showing quantitative results. n = 6.

**(D)** Real-time quantitative PCR (qPCR) analysis of OTUD5 mRNA levels in myocardial tissues of OTUD5fl/y and OTUD5CKO mice. n = 6.

Statistical significance was defined as: *****P*< 0.0001.


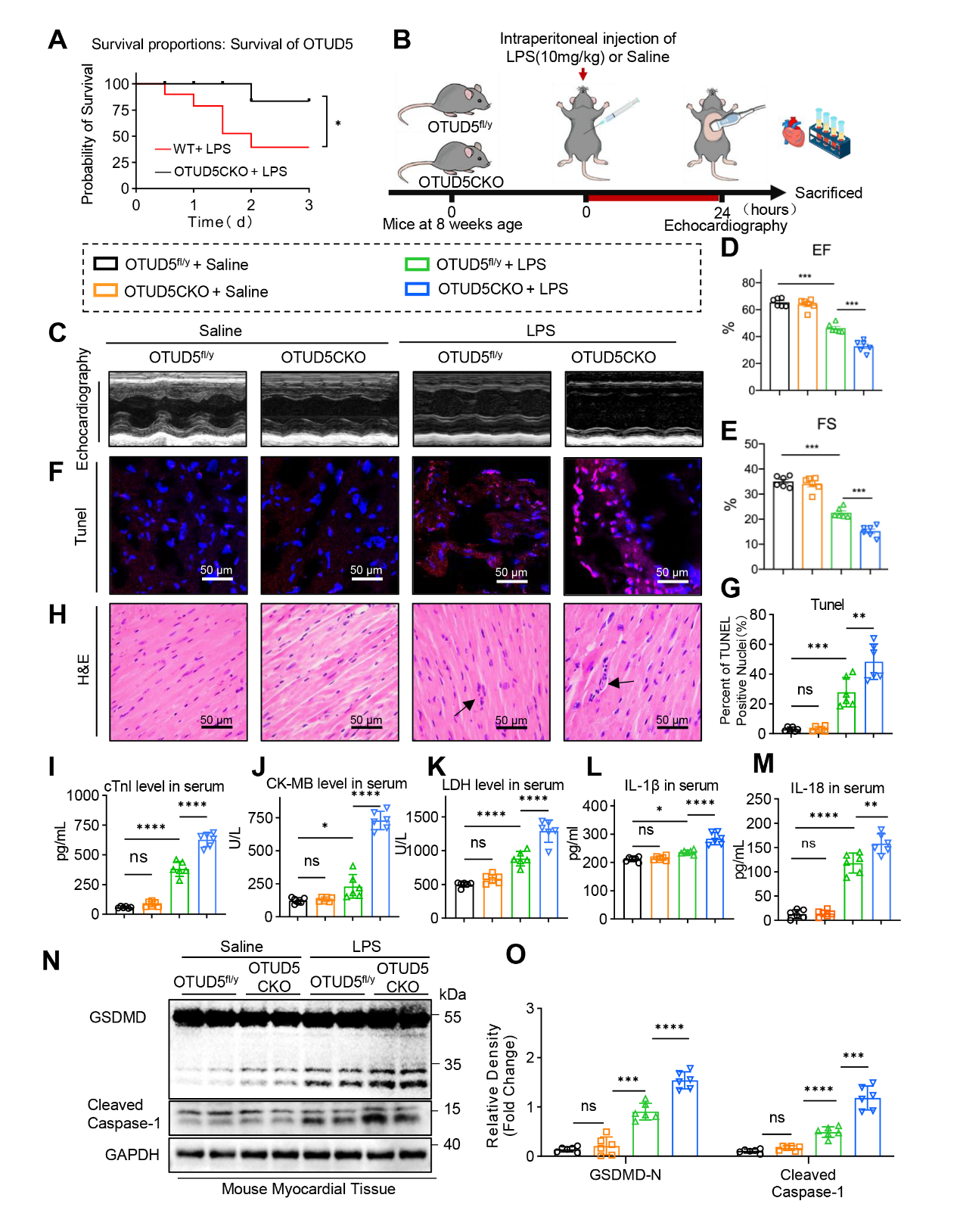
**Supplementary Figure S5**

**Supplementary Figure S5. Cardiomyocyte-specific knockout of OTUD5 exacerbates LPS-induced septic myocardial injury and pyroptosis**

**(A)** Survival curves of wild-type (WT) mice and OTUD5 conditional knockout (OTUD5CKO) mice following intraperitoneal injection of LPS (10 mg/kg) to induce septic cardiomyopathy. n = 10.

**(B)** Experimental workflow for the LPS-induced septic cardiomyopathy mouse model.

**(C)** M-mode echocardiography images of mouse hearts from each group.

**(D)** Quantitative analysis of left ventricular ejection fraction (EF %). n = 6.

**(E)** Quantitative analysis of left ventricular fractional shortening (FS %). n = 6.

**(F-G)** TUNEL staining to detect cardiomyocyte pyroptosis (apoptotic nuclei labeled with red fluorescence), with corresponding statistical analysis of pyroptotic cell proportion. n = 6.

**(H)** Representative H&E-stained images of myocardial tissue from each group.

**(I)** Serum cardiac troponin T (cTnT) levels from each group in mice. n = 6.

**(J)** Serum creatine kinase-MB (CK-MB) activity from each group in mice. n = 6.

**(K)** Serum lactate dehydrogenase (LDH) release from each group in mice. n = 6.

**(L)** Myocardial tissue supernatant IL-1β content. n = 6.

**(M)** Myocardial tissue supernatant IL-18 content. n = 6.

**(N-O)** Western blotting analysis of GSDMD-N and Cleaved Caspase-1 protein levels in mouse myocardium, with corresponding statistical bar graphs. n = 6.

Statistical significance was defined as: *P < 0.05, ***P < 0.001 and ****P < 0.0001. The abbreviation "NS" indicates no statistical significance (P > 0.05).​

**Supplementary Figure S6**

**
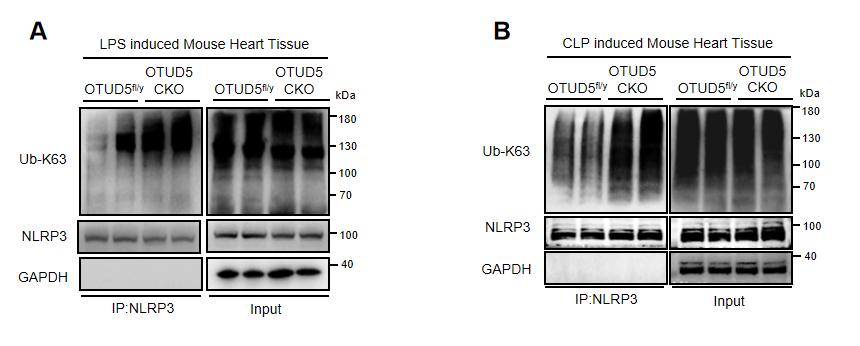
**

**Supplementary Figure S6. The K63 ubiquitination level of NLRP3 was significantly increased in LPS-induced and CLP-induced myocardial tissue of OTUD5 knockout mice. (A-B)** Immunoprecipitation of NLRP3 in LPS / CLP-induced cardiac tissues of OTUD5^fl/y^ and OTUD5CKO mice. Ubiquitinated NLRP3 was detected by immunoblotting using an Ub-K63 antibody to clarify the K63 ubiquitination level of NLRP3 regulated by OTUD5.

**
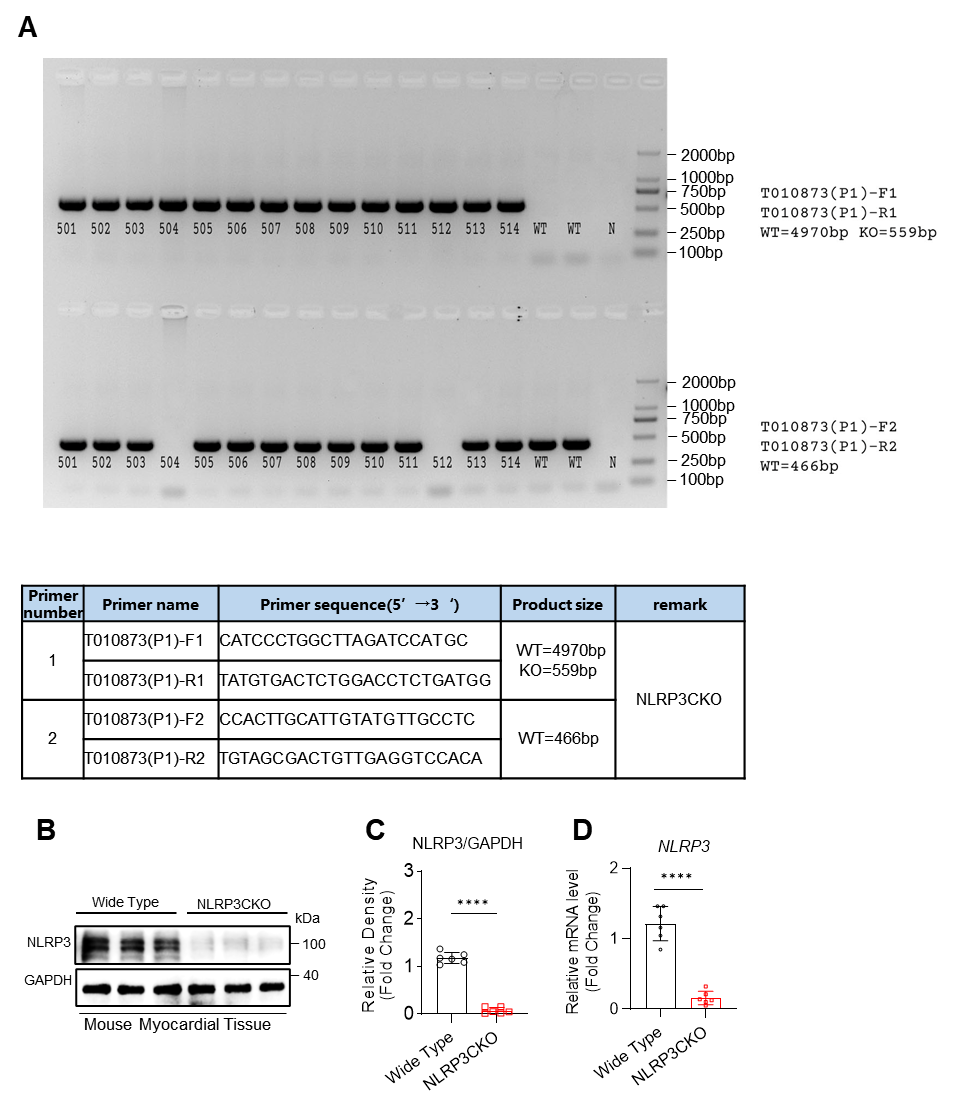
Supplementary Figure S7**

**Supplementary Figure S7.** **Generation and validation NLRP3 knockout mice. (A)** Genotyping of mouse tail DNA to verify NLRP3 knockout using primers 1, 2. NLRP3CKO mice exhibit only the bands corresponding to primers 1 (559 bp).

**(B–C)** Western blot analysis of NLRP3 protein expression in myocardial tissues of NLRP3CKO and Wide type mice, along with a corresponding bar graph showing quantitative results. n = 6.

**(D)** Real-time quantitative PCR (qPCR) analysis of OTUD5 mRNA levels in myocardial tissues of NLRP3CKO and Wide type mice. n = 6.

Statistical significance was defined as: ****P< 0.0001.

**Supplementary Figure S8**

**
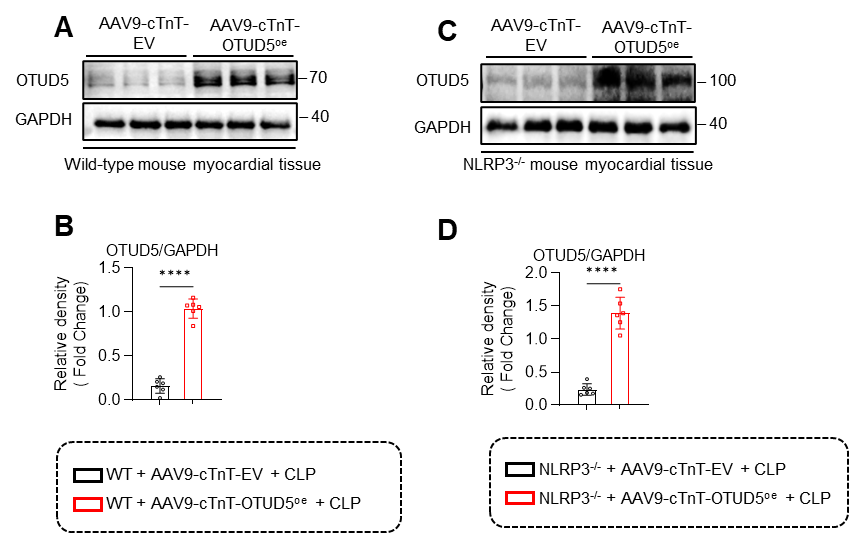
**

**Supplementary Figure S8. The mice underwent tail vein injections with either AAV9-cTnT-OTUD5 or an empty vector (EV) control. (A, C)** Western blot was used to detect the expression of OTUD5 protein in myocardial tissue of wild-type and NLRP3^-/-^ mice after AAV overexpression.

**(B, D)** Statistical analysis of OTUD5 protein levels in wild-type and NLRP3^-/-^ mice. n= 6.
